# Effect of Massage Therapy on Vital Signs and GCS Scores of ICU Patients: A Randomized Controlled Clinical Trial

**DOI:** 10.5812/traumamon.17031

**Published:** 2014-08-01

**Authors:** Amir Vahedian-Azimi, Abbas Ebadi, Mohammad Asghari Jafarabadi, Soheil Saadat, Fazlollah Ahmadi

**Affiliations:** 1 Trauma Research Center, Faculty of Nursing, Baqiyatallah University of Medical Sciences, Tehran, IR Iran; 2 Behavioral Sciences Research Center, Faculty of Nursing, Baqiyatallah University of Medical Sciences, Tehran, IR Iran; 3 Road Traffic Injury Prevention Research Center, Department of Statistics and Epidemiology, Tabriz University of Medical Sciences, Tabriz, IR Iran; 4 Sina Trauma and Surgery Research Center, Tehran University of Medical Sciences, Tehran, IR Iran; 5 Department of Nursing, Faculty of Medical Sciences, Tarbiat Modares University, Tehran, IR Iran

**Keywords:** Massage, Vital Signs, Glasgow Coma Scale, Intensive Care Unit

## Abstract

**Background:**

Unalleviated complications related to hospitalization, including stress, anxiety, and pain, can easily influence different structures, like the neural system, by enhancing the stimulation of sympathetic nervous pathways and causing unstable vital signs and deterioration in the level of consciousness.

**Objectives:**

The purpose of this study was to determine the effects of massage therapy by family members on vital signs and Glasgow Coma Scale Score (GCS) of patients hospitalized in the Intensive Care Unit (ICU).

**Patients and Methods:**

This randomized controlled clinical trial was conducted at the ICU of the Shariati Hospital during 2012; 45 ICU patients and 45 family members in the experimental group and the same number of patients and family members in the control group were consecutively selected . The data collection instrument consisted of two parts. The first part included demographic data (age, marital status and Body Mass Index) and the second part included a checklist to record the patient’s vital signs (systolic blood pressure (SBP), diastolic blood pressure (DBP), respiratory rate (RR), pulse rate (PR)) and GCS. All measurements were done at the same time in both groups before the intervention (full body massage therapy), and 1 hour, 2 hours, 3 hours, and 4 hours after intervention. The patients were provided with a 60-minute full body massage The massage protocol included static, surface tension, stretching, superficial lymph unload, transverse friction, and myofacial releasing techniques.

**Results:**

Significant differences were observed between experimental and control groups in the SBP at 1 hour, SBP 2 hours, and SBP 3 hours, and also in GCS at 1 hour to GCS at 4 hours (P < 0.05). Multivariate analysis revealed a significant difference between experimental and control groups in SBP at all time points (P < 0.05).

**Conclusions:**

Massage via family members had several positive effects on the patients’ clinical conditions, and therefore, it should be recognized as one of the most important clinical considerations in hospitalized patients.

## 1. Background

Hospitalization in the intensive care unit (ICU) and complications related to hospitalization, including stress, anxiety, pain, and fear from an unknown environment, can cause hemodynamic instabilities (increased blood pressure, pulse rate (PR) and respiratory rate (RR)), and deterioration of the consciousness levels and Glasgow Coma Scale (GCS) scores (1-3). The critical care environment represents a multifaceted experience for each patient, which may include anxiety due to the nature of the surroundings and their medical or surgical condition. Anxiety under such conditions may not only be manifested at a psychological level, but may also be detrimental to the physical well-being of the patient, which may, in turn, lead to a prolonged sojourn in critical care (4).

Vital signs are sensitive to pain, stress and anxiety, and are affected by nervous system function (5). The unalleviated previously mentioned symptoms can easily affect different body systems, including cardiovascular, pulmonary, and endocrine, as well as stimulate sympathic nerves and cerebral pathways. As a result, they generate instabilities in vital signs and deterioration in consciousness levels (6). In recent years, alternative and complementary therapies, including music therapy, relaxation, guided imagery, reflexology, herbal medicine, hypnosis, therapeutic touch, and therapeutic massage, have been used to decrease the mentioned side effects (7-9).

Massage therapy is an ancient therapeutic technique that has been utilized in most major healing traditions. Recent interests in the role of massage therapy, as a supplement to conventional medical therapy, have resulted in an extensive number of clinical trials, many of which have shown improvements in hemodynamic and nervous system functions (1-3). Massage causes a feeling of wellbeing, relaxation and comfort (10). The use of lubricant gel is also recommended to facilitate the massage. Almond oil has been suggested as a lubricant by some (11).

Several studies have focused on the role of family members in the ICU setting in providing care to patients hospitalized in ICUs (12-16). Furthermore, an increasing number of studies have probed the relationship between families and nurses (17). In the past two decades, there has been a movement to promote and motivate family members of patients as partners in nursing care and family-centered care (FCC) (18). Symptoms of anxiety, stress, and depression are common in families of ICU patients (19); and posttraumatic stress symptoms with adverse effects on the quality of life were found in 34% of family members 3 months after ICU discharge (20-22). These results have encouraged the conduction of extensive studies on the importance of family involvement in treating patients hospitalized in the ICU. Families need information, reassurance, and support, and they also need to be near the patient (23). To fulfill such needs, FCC has been developed in ICUs. The FCC is a philosophy of care that regards the patient and family as a unit of care, rather than having the patient as the sole focus of care. It is seen as a way to optimize patient outcomes through a collaborative partnership process with healthcare services (24).

Family members are important, as the family is included in care when a person becomes critically ill in an ICU (15). Family feedback on the interventions provided by nurses in the healthcare setting is positive among patients who are mentally ill, critically ill, or elderly (14). The FCC is an approach to medical care originally rooted in the belief that optimal health outcomes are obtained when family members of patients play an active role in providing physical, psychological, emotional, social, and developmental support. Family support activities and programs are conducted with the purpose of helping families cope with the stress of having a patient in intensive care and supporting the family, as they join in the care of their patient (13).

## 2. Objectives

This study aimed to determine the effects of massage therapy by family members on vital signs and GCS of patients hospitalized in the ICU.

## 3. Patients and Methods

### 3.1. Study Design

This was a triple-blinded randomized controlled clinical trial study.

### 3.2. Samples and Setting

**Figure 1.**
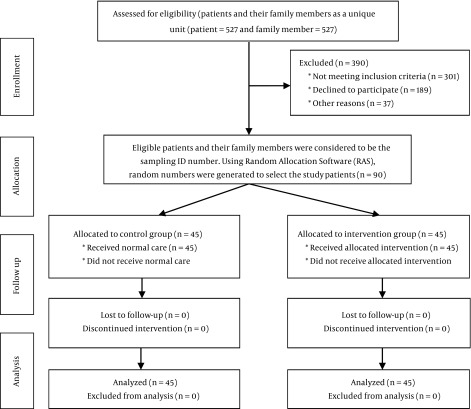
Flow Chart of Sampling Process

The study was conducted in the General Intensive Care Unit (GICU) of the Shariati Hospital in Tehran, during 2012. The patients in the study were patients with prolonged hospitalization (more than 10 days of hospitalization). Inclusion criteria before the intervention were: more than 10 days of hospitalization; hemodynamic stability; having a GCS of 7 - 12 (3); intracranial pressure less than 20 mmHg; and no limitation or contraindication for changes of body position (i.e. active bleeding or flail chest) or body massage (such as severe burns, severe dyspnea, fever, etc.). All parts of the study were reviewed according to the Consolidated Standards for Reporting Trials (CONSORT) statement (Figure 1) (25, 26). In the first phase, convenience sampling method was used. All patients with prolonged hospitalization who met the inclusion criteria were recruited. The sample size was determined using the information obtained from a pilot study with 10 patients and 10 family members. By considering a confidence interval of 95% and a power of 80%, a sample size of at least 40 cases was determined for patients and family members. To ensure a sufficient number of family members and patients after any possible attrition from the study process, a total of 45 qualified patients and family members were asked to participate. In other words, from the 527 eligible patients and their 527 eligible family members, 90 were included in the study. Then, in the second step, random allocation was conducted using a random assignment software (RAS). Afterwards, 45 patients and 45 family members were placed in the experimental group and equal numbers were selected for the control group. For allocation of the patients, a computer-generated list of random numbers was used. Patients were randomly assigned to one of the two treatment groups following simple randomization procedures (computerized random numbers). Block randomization was performed by a computer generated random number list prepared by an expert statistician who had no clinical involvement in the trial. Patients with prolonged hospitalization were categorized based on the treatment procedure and admission date.

### 3.3. Ethical Considerations

The present study was registered at Clinical Trials. gov with identifier NCT01909882 and was registered at the Research Committee of Baqiyatallah University of Medical Sciences, number 388. Informed consent was obtained prior to the study.

### 3.4. Intervention

Family members received massage training, and were tested afterwards. After passing the test, they were allowed to massage the patient. The training sessions of family members were conducted individually in two 2-hour sessions (first session for educating and the second for practicing) on a mannequin in the practice room at the hospital. All the training sessions were conducted by the first researcher who was certified in massage therapy. Then, following the confirmation of prolonged hospitalization, each patient in the intervention group was massaged by a family member in a private atmosphere during a 60-minute session. Almond oil was used for effleurage and massage facilitation. Back, shoulder, deltoid muscles, front and posterior parts of the legs, arms, forearms, front and back parts of thighs, palms and fingers, metatarsus, front and back of feet and toes, belly and chest, axillary and neck muscles of the patients were massaged. Massage techniques included static massage, surface tension techniques, stretching massage, superficial lymph unload, transverse friction techniques, and myofacial releasing techniques (11). All the massage sessions were conducted during morning shifts. Areas with inflammation, petechiae, ecchymosis, subcutaneous hemorrhage, wounds or edema were not massaged. During the massage therapy, sustainability of general, hemodynamic and airway conditions were considered. No intervention was provided for the control group, and they received just the routine care of the unit. The vital signs and GCS score were measured similarly in intervention and control groups.

### 3.5. Data Collection

Study subjects from the ICU were randomly allocated into the groups by RAS (Figure 1). The data collection consisted of two parts. The first part included demographic data (age, marital status and body mass Index (BMI)). The second part included a checklist to record the patient’s vital signs (systolic blood pressure (SBP), diastolic blood pressure (DBP), RR, and PR). All measurements were conducted simultaneously in both groups before the intervention, and 1 hour, 2 hours, 3 hours, and 4 hours after intervention. We used the practical version of the GCS . Each score report was examined by one anesthesiologist for verification. The Kappa agreement test between the first researcher and anesthesiologist was significant (0.9). 

### 3.6. Data Analysis

All analyses were performed using SPSS 15.0 (SPSS Inc., Chicago, IL). Frequency (percent), mean (Standard Deviation) were presented for qualitative and quantitative variables, respectively. For the GCS, due to non-normality of the variable, the median (Quartile 1 to Quartile 3) was presented. The normality of the study variables was tested by the Kolmogorov-Smirnov one-sample test. Normality was confirmed for the SBP, DBP, PR and RR. Therefore, repeated measurements of analysis of variance (ANOVA) were performed to assess the changes of the mean values over time for the experimental and control groups, followed by the Sidak post hoc test. The assumption of the sphericity of the covariance matrix was evaluated using Mauchly's Test and, depending on the results of this test, P values were presented based on the Greenhouse-Geiser correction. In addition, Hotteling’s T2 tests evaluated the differences between experimental and control groups at all time points, followed by independent samples t-tests for investigating the differences between the experimental and control groups separately, at each time point. Normality was not confirmed for GCS. Therefore, Friedman’s test was performed to assess the changes of the mean values over time for the experimental and control groups. In addition, Mann-Whitney U test was used to compare experimental and control groups separately at each time point. The comparisons of background variables including age, sex, marital status and BMI categories were investigated between the two groups using t-test, Chi-Squared Test or Fisher’s Exact Test. P values < 0.05 were considered significant.

## 4. Results

No significant differences were observed between experimental and control groups in demographic variables of age, sex, marital status and BMI categories (Table 1). From the perspective of sex, 60 percent and 70 percent of experimental and control groups, were male, respectively, and no significant correlation was found between experimental and control groups. Concerning the BMI, Fisher’s exact test showed that the majority of patients were in the normal range (18.51-25.00) Independent sample t-test showed that the average age of the patients in the experimental and control groups was 61.2 ± 10.17 years and 59.9 ± 9.23 years, respectively. The results of the Kolmogorov-Smirnov one-sample test confirmed the normality for DBP, PR and RR but not for the GCS (all P < 0.05).

The comparisons of all variables between the experimental and control groups are listed in Tables 2 and 3. According to data presented in the aforementioned tables, significant differences were observed between experimental and control groups in SBP 1 (121.97 ± 12.21 vs. 128.06 ± 13.05, P < 0.048), SBP 2 (121.37 ± 11.37 vs. 128.20 ± 12.21, P < 0.018), and SBP 3 (120.51 ± 10.60 vs. 127.57 ± 12.12, P < 0.012), and similarly in the GCS 1 (median = 8 and Q1-Q3 = 8-9 vs. median = 7 and Q1-Q3 = 7-8, P < 0.001), GCS 2 (median = 8 and Q1-Q3 = 8-9 vs. median = 7 and Q1-Q3 = 7-8, P < 0.001), GCS 3 (median = 9 and Q1-Q3 = 9-9 vs. median = 7 and Q1-Q3 = 7-8, P < 0.001), and GCS 4 (median = 9 and Q1-Q3 = 9-10 vs. median = 7 and Q1-Q3 = 7-8, P < 0.001). Differences were not significant for other variables (P > 0.05, Table 2). Multivariate analysis revealed a significant difference between experimental and control groups in SBP at all-time points (P < 0.05). Moreover, the magnitude of the changes over the time points was seen. The changes for all study variables were significant over time in the experimental group (P < 0.05). However, in the control group, the results revealed no significant changes over time (P > 0.05). 

**Table 1.** Summary Statistics and the Results of the Tests for Comparing the Groups for Background Variables^a^

	Frequency (%)	P Value
**Sex (Male)**		0.615 ^b^
Exp	22 (60)	
Cont	24 (70)	
**Marital (Married)**		1.000 ^b^
Exp	34 (97.1)	
Cont	35 (100)	
**BMI Category, kg/m^2^**		0.854 ^c^
**≤ 18.50**		
Exp	1 (2.9)	
Cont	0 (0.0)	
**18.51-25.00**		
Exp	15 (42.9)	
Cont	19 (54.3)	
**25.01-30.00**		
Exp	18 (51.4)	
Cont	12 (34.3)	
**+30.01**		
Exp	1 (2.9)	
Cont	4 (11.4)	

^a^ Abbreviations: BMI, body mass index; Cont, control; Exp, experimental.

^b^ Based on Chi-Squared test.

^c^ Based on Fisher’s exact test.

**Table 2.** Summary Statistics and the Results of the Tests for Comparing the Repeated Measurements Within Groups and Comparing Groups for Study Variables ^a^

	Mean ± SD	P value ^b^	Within Cont P ^c^	Within Exp P ^d^	Between Group P ^e^
**SBP, mm Hg**		0.105	0.391	< 0.001	0.046
Exp	122.77 ± 12.92				
Cont	127.94 ± 13.40				
**SBP 1**		0.048			
Exp	121.97 ± 12.21				
Cont	128.06 ± 13.05				
**SBP 2**		0.018			
Exp	121.37 ± 11.43				
Cont	128.20 ± 12.21				
**SBP 3**		0.012			
Exp	120.51 ± 10.60				
Cont	127.57 ± 12.12				
**SBP 4**		0.132			
Exp	123.31 ± 12.30				
Cont	127.94 ± 13.09				
**DBP, mmHg**		0.239	0.148	0.020	0.346
Exp	75.34 ± 11.60				
Cont	72.34 ± 9.42				
**DBP 1**		0.280			
Exp	74.80 ± 11.16				
Cont	72.14 ± 9.15				
**DBP 2**		0.376			
Exp	74.57 ± 10.90				
Cont	72.40 ± 9.43				
**DBP 3**		0.697			
Exp	73.26 ± 8.28				
Cont	72.43 ± 9.40				
**DBP 4**		0.292			
Exp	75.09 ± 11.08				
Cont	72.46 ± 9.55				
**PR**		0.878	0.436	< 0.001	0.803
Exp	78.14 ± 11.61				
Cont	77.71 ± 11.74				
**PR 1**		0.876			
Exp	77.40 ± 11.08				
Cont	77.83 ± 11.85				
**PR 2**		0.673			
Exp	76.86 ± 10.52				
Cont	78.00 ± 12.00				
**PR 3**		0.433			
Exp	75.89 ± 9.84				
Cont	77.91 ± 11.59				
**PR 4**					
Exp	77.77 ± 11.58	0.942			
Cont	77.97 ± 11.37				
**RR**			0.528	0.003	0.211
Exp	17.71 ± 3.59	0.442			
Cont	18.40 ± 3.82				
**RR 1**					
Exp	17.51 ± 3.37	0.240			
Cont	18.51 ± 3.67				
**RR 2**					
Exp	17.37 ± 3.29	0.187			
Cont	18.49 ± 3.69				
**RR3**					
Exp	16.91 ± 2.86	0.059			
Cont	18.46 ± 3.78				
**RR 4**					
Exp	17.63 ± 3.46	0.276			
Cont	18.54 ± 3.50				

^a^ Abbreviations: Cont, control; Exp, experimental; DBP, diastolic blood pressure; PR, pulse rate; RR, respiratory rate; SBP, systolic blood pressure.

^b^ P value based on independent samples t-test for comparison of Exp and Cont groups at each time point.

^c^ P value based on repeated measures ANOVA for testing within the control group the changes over time. Dependent on the results of Mauchly’s test, P values presented are based on the Greenhouse-Geiser test.

^d^ P value based on repeated measures ANOVA for testing within the experimental group the changes over time. Dependent on the results of Mauchly’s test, P values presented are based on the Greenhouse-Geiser test.

^e^ P value based on Hotelling T2 for overall comparison of the experimental and control groups.

**Table 3.** Summary Statistics and the Results of the Tests for Comparing the Repeated Measures Within Groups and Comparing Groups for Study Variables ^a^

	Median	(Q1-Q3)	P Value ^b^	Within Cont P ^c^	Within Exp P ^d^
**GCS @**			0.278	0.548	< 0.001
Exp	8	(7-8)			
Cont	7	(7-8)			
**GCS 1 @**			< 0.001		
Exp	8	(8-9)			
Cont	7	(7-8)			
**GCS 2 @**			< 0.001		
Exp	9	(8-9)			
Cont	7	(7-8)			
**GCS 3 @**			< 0.001		
Exp	9	(9-9)			
Cont	7	(7-8)			
**GCS 4 @**			< 0.001		
Exp	9	(9-10)			
Cont	7	(7-8)			

^a^ Abbreviations: Cont, control; Exp, experimental; GCS, Glasgow Coma Scale; Q, quartile.

^b^ P value based on Mann-Whitney U test for comparison of the experimental and control groups at each time point.

^c^ P value based on Friedman’s test for testing changes over time within the control group.

^d^ P value based on Friedman’s test for testing changes over time within the experimental group.

Note that for GCS on Hotelling T2 test was performed for overall comparison of experimental and control groups.

## 5. Discussion

The results of this study showed that full body massage therapy reduces SBP and increases the level of consciousness and is consistent with other studies (4-6). This result underlines the mechanism of parasympathic activation following full body message, which resulted in decreasing physiological responses. This decrease in SBP may indicate that the patients were more relaxed (4, 11). Full body massage may distract the patient and consequently reduce anxiety (27), and can lead to a decrease in BP and induce the patients a sense of comfort and relaxation (28), which may then result in the secretion of endorphins (29); vessels become more dilated, the blood flow increases within the superficial vessels of body (30) and BP will decrease. It can be expressed that, although an extensive review of the literature demonstrated that massage therapy could decrease RR, PR, and BP, in our study these variables did not statistically decrease over time. 

The results of the present study demonstrated that full body massage therapy by family members increases the level of consciousness of patients. Although few studies have been conducted on this subject, the significance of family participation in providing care to hospitalized patients in the ICU has been noticed by previous studies. Optimal health outcomes are achieved when patients’ family members play an active role in providing physical, psychological, emotional, social, and developmental care for their patients (13,31). The possible reason for the effectiveness of family members’ involvement can be explained through the transfer of their emotion to patients. The ICU is an unknown environment with multiple unfamiliar subjects, and patients try to find more familiar things while hospitalized there. Family members, in the context of their emotional support, are excellent motivators. 

The study was carried out to assess the impact of family members’ massage therapy on vital signs and GCS score of patients in the ICU. The results of this study revealed that complementary approaches, such as massage therapy, have a beneficial effect on improving vital signs and GCS score of these patients. Furthermore, involvement of family members should be recognized as one of the most important clinical considerations for all hospitalized patients, especially patients in the ICU, as family members are an effective bridge between patients and medical personnel.
